# Supplementary material for: A Novel Urine Exosomal lncRNA Assay to Improve the Detection of Prostate Cancer at Initial Biopsy: A Retrospective Multicenter Diagnostic Feasibility Study
Source: Cancers (Basel). 2021 Aug 13;13(16):4075. doi: 10.3390/cancers13164075 (PMC8393262; doi:10.3390/cancers13164075)
Supplement: Supplementary file 1 [file cancers-13-04075-s001.zip › cancers-1325555-supplementary.pdf]

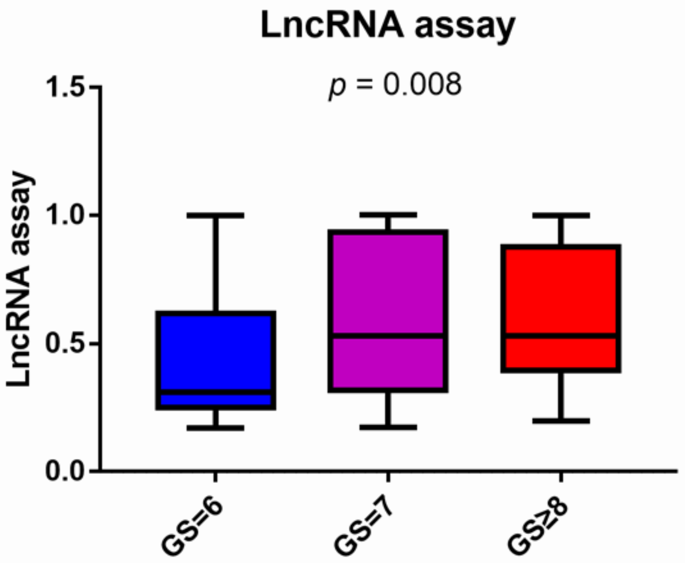

Suplementary Figure S1 The lncRNA assay was corelated with the malignancy grade of prostate cancer (PCa)

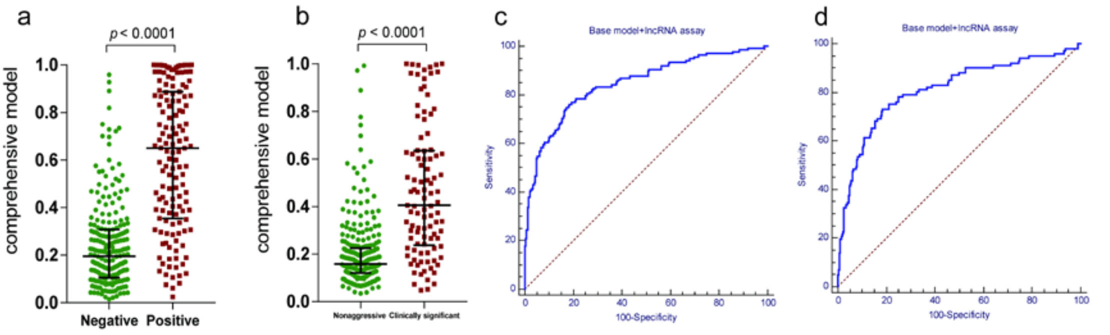

Supplementary Figure S2 The diagnosis performance of comprehensive model. a, The probability were significantly elevated in PCa patients than negative biopsy patients. b, The probability were significantly elevated in clinically significant PCa patients than non-aggressive disease patients. ROC analysis shows the diagnostic power of comprehensive model in PCa (c, AUC:0.846) and clinically significant PCa (e, AUC:0.811).

Table S1 The list of primers and probes

NO;	Gene name	Primer sequence	Probe sequence
1	*MALAT1-F*	AGTTTTAAGCAGTCGTATTTGTGA	VIC-CCTCCCACCACCAGAAATG-MGB
	MALAT1-R	GTCACTGATTTTAAGGTTGCATCT	
2	*PCA3-F*	AAGAAATAGCAAGTGCCGAGAA	VIC-CAGATCTTCCTGGTCTCCCTC-MGB
	PCA3-R	CATCAGGTCCTTCCCACCAT	
3	*ACTB-F*	TGCGTTACACCCTTTCTT	FAM-CAAAACCTAACTTGCGCAGAAAAC-MGB
	ACTB-R	CTGTCACCTTCACCGTTC	
